# Supplementary material for: The Assessment of White Matter Integrity Alteration Pattern in Patients with Brain Tumor Utilizing Diffusion Tensor Imaging: A Systematic Review
Source: Cancers (Basel). 2023 Jun 24;15(13):3326. doi: 10.3390/cancers15133326 (PMC10341056; doi:10.3390/cancers15133326)
Supplement: Supplementary file 1 [file cancers-15-03326-s001.zip › cancers-2438382-supplementary.pdf]

Supplementary Material

The Assessment of White Matter Integrity Alteration Pattern in Patients with Brain Tumor Utilizing Diffusion Tensor Imaging: A Systematic Review

Aiman Abdul Manan [1], Noorazrul Azmie Yahya [2], Nur Hartini Mohd Taib [3,4], Zamzuri Idris [3,5] and Hanani Abdul Manan [1,6,*]

Supplementary Data

TABLE S1: PRISMA 2020 Checklist

Section and Topic	Item #	Checklist item	Location where item is reported (page no)
**TITLE**			
Title	1	Identify the report as a systematic review.	1
**ABSTRACT**			
Abstract	2	See the PRISMA 2020 for Abstracts checklist.	-
**INTRODUCTION**			
Rationale	3	Describe the rationale for the review in the context of existing knowledge.	2
Objectives	4	Provide an explicit statement of the objective(s) or question(s) the review addresses.	2
**METHODS**			
Eligibility criteria	5	Specify the inclusion and exclusion criteria for the review and how studies were grouped for the syntheses.	3
Information sources	6	Specify all databases, registers, websites, organisations, reference lists and other sources searched or consulted to identify studies. Specify the date when each source was last searched or consulted.	3
Search strategy	7	Present the full search strategies for all databases, registers and websites, including any filters and limits used.	4
Selection process	8	Specify the methods used to decide whether a study met the inclusion criteria of the review, including how many reviewers screened each record and each report retrieved, whether they worked independently, and if applicable, details of automation tools used in the process.	3-4
Data collection process	9	Specify the methods used to collect data from reports, including how many reviewers collected data from each report, whether they worked independently, any processes for obtaining or confirming data from study investigators, and if applicable, details of automation tools used in the process.	3-4
Data items	10a	List and define all outcomes for which data were sought. Specify whether all results that were compatible with each outcome domain in each study were sought (e.g. for all measures, time points, analyses), and if not, the methods used to decide which results to collect.	3-5
	10b	List and define all other variables for which data were sought (e.g. participant and intervention characteristics, funding sources). Describe any assumptions made about any missing or unclear information.	3-5

Section and Topic	Item #	Checklist item	Location where item is reported (page no)
Study risk of bias assessment	11	Specify the methods used to assess risk of bias in the included studies, including details of the tool(s) used, how many reviewers assessed each study and whether they worked independently, and if applicable, details of automation tools used in the process.	3-5
Effect measures	12	Specify for each outcome the effect measure(s) (e.g. risk ratio, mean difference) used in the synthesis or presentation of results.	3-5
Synthesis methods	13a	Describe the processes used to decide which studies were eligible for each synthesis (e.g. tabulating the study intervention characteristics and comparing against the planned groups for each synthesis (item #5)).	Figure 1
	13b	Describe any methods required to prepare the data for presentation or synthesis, such as handling of missing summary statistics, or data conversions.	4-6,<br>Figure 1, Table 2,3,4,5
	13c	Describe any methods used to tabulate or visually display results of individual studies and syntheses.	4-6
	13d	Describe any methods used to synthesize results and provide a rationale for the choice(s). If meta-analysis was performed, describe the model(s), method(s) to identify the presence and extent of statistical heterogeneity, and software package(s) used.	4-6
	13e	Describe any methods used to explore possible causes of heterogeneity among study results (e.g. subgroup analysis, meta-regression).	4-6, Table 4,5
	13f	Describe any sensitivity analyses conducted to assess robustness of the synthesized results.	-
Reporting bias assessment	14	Describe any methods used to assess risk of bias due to missing results in a synthesis (arising from reporting biases).	4
Certainty assessment	15	Describe any methods used to assess certainty (or confidence) in the body of evidence for an outcome.	-
**RESULTS**			
Study selection	16a	Describe the results of the search and selection process, from the number of records identified in the search to the number of studies included in the review, ideally using a flow diagram.	Figure 1
	16b	Cite studies that might appear to meet the inclusion criteria, but which were excluded, and explain why they were excluded.	3
Study characteristics	17	Cite each included study and present its characteristics.	Table 2
Risk of bias in studies	18	Present assessments of risk of bias for each included study.	3
Results of individual studies	19	For all outcomes, present, for each study: (a) summary statistics for each group (where appropriate) and (b) an effect estimate and its precision (e.g. confidence/credible interval), ideally using structured tables or plots.	Table 2,3,4,5,6
Results of syntheses	20a	For each synthesis, briefly summarise the characteristics and risk of bias among contributing studies.	-
	20b	Present results of all statistical syntheses conducted. If meta-analysis was done, present for each the summary estimate and its precision (e.g. confidence/credible interval) and measures of statistical heterogeneity. If comparing groups, describe the direction of the effect.	Table 2,3,4,5,6
	20c	Present results of all investigations of possible causes of heterogeneity among study results.	Table 2,3,4,5,6
	20d	Present results of all sensitivity analyses conducted to assess the robustness of the synthesized results.	Table 2,3,4,5,6

Section and Topic	Item #	Checklist item	Location where item is reported (page no)
Reporting biases	21	Present assessments of risk of bias due to missing results (arising from reporting biases) for each synthesis assessed.	5-21
Certainty of evidence	22	Present assessments of certainty (or confidence) in the body of evidence for each outcome assessed.	5-21
**DISCUSSION**			
Discussion	23a	Provide a general interpretation of the results in the context of other evidence.	4-23
	23b	Discuss any limitations of the evidence included in the review.	23
	23c	Discuss any limitations of the review processes used.	23-24
	23d	Discuss implications of the results for practice, policy, and future research.	23-24
**OTHER INFORMATION**			
Registration and protocol	24a	Provide registration information for the review, including register name and registration number, or state that the review was not registered.	4
	24b	Indicate where the review protocol can be accessed, or state that a protocol was not prepared.	4
	24c	Describe and explain any amendments to information provided at registration or in the protocol.	-
Support	25	Describe sources of financial or non-financial support for the review, and the role of the funders or sponsors in the review.	24
Competing interests	26	Declare any competing interests of review authors.	24
Availability of data, code and other materials	27	Report which of the following are publicly available and where they can be found: template data collection forms; data extracted from included studies; data used for all analyses; analytic code; any other materials used in the review.	24, Supplementary Material

[Ref] Page, M.J.; McKenzie, J.E.; Bossuyt, P.M.; Boutron, I.; Hoffmann, T.C.; Mulrow, C.D.; The PRISMA 2020 statement: an updated guideline for reporting systematic reviews. *BMJ* **2021**;372:n71. doi: 10.1136/bmj.n7

Table S2: PRISMA 2020 for Abstract Checklist

Section and Topic	Item #	Checklist item	Reported (Yes/No)
**TITLE**			
Title	1	Identify the report as a systematic review.	Yes
**BACKGROUND**			
Objectives	2	Provide an explicit statement of the main objective(s) or question(s) the review addresses.	Yes
**METHODS**			
Eligibility criteria	3	Specify the inclusion and exclusion criteria for the review.	No
Information sources	4	Specify the information sources (e.g. databases, registers) used to identify studies and the date when each was last searched.	No
Risk of bias	5	Specify the methods used to assess risk of bias in the included studies.	Yes
Synthesis of results	6	Specify the methods used to present and synthesise results.	Yes
**RESULTS**			
Included studies	7	Give the total number of included studies and participants and summarise relevant characteristics of studies.	Yes

Section and Topic	Item #	Checklist item	Reported (Yes/No)
Synthesis of results	8	Present results for main outcomes, preferably indicating the number of included studies and participants for each. If meta-analysis was done, report the summary estimate and confidence/credible interval. If comparing groups, indicate the direction of the effect (i.e. which group is favoured).	Yes
**DISCUSSION**			
Limitations of evidence	9	Provide a brief summary of the limitations of the evidence included in the review (e.g. study risk of bias, inconsistency and imprecision).	No
Interpretation	10	Provide a general interpretation of the results and important implications.	Yes
**OTHER**			
Funding	11	Specify the primary source of funding for the review.	No (ND)
Registration	12	Provide the register name and registration number.	No (ND)

[Ref] Page, M.J.; McKenzie, J.E.; Bossuyt, P.M., Boutron, I.; Hoffmann, T.C.; Mulrow, C.D.; The PRISMA 2020 statement: an updated guideline for reporting systematic reviews. *BMJ* **2021**;372:n71. doi: 10.1136/bmj.n71

Table S3 Advanced Search Keyword chain in Pubmed,Scopus and Wos

Database	Search String	Articles found
Phase 1		
Pubmed	(((((((diffusion tensor imaging) OR (diffusion tensor tractography)) OR (diffusion tensor)) OR (tractography)) OR (fiber tracking)) OR (fibre tracking)) AND (((((brain tumor) OR (brain tumour)) OR (brain lesion)) OR (brain cancer)) OR (brain neoplasm))) AND (((((((((white matter tract integrity) OR (white matter tract morphology)) OR (white matter tract microstructure)) OR (white matter tract topology)) OR (white matter tract characterization)) OR (white matter tract characterisation)) OR (white matter criteria)) OR (white matter tract alteration)) OR (white matter tract changes))	921
Scopus	((TITLE-ABS-KEY (diffusion AND tensor AND imaging) OR TITLE-ABS-KEY (diffusion AND tensor AND tractography) OR TITLE-ABS-KEY (diffusion AND tensor) OR TITLE-ABS-KEY (tractography) OR TITLE-ABS KEY (fiber AND tracking) OR TITLE-ABS-KEY (fibre AND tracking))) AND ((TITLE-ABS-KEY (brain AND tumor) OR TITLE-ABS-KEY (brain AND tumour) OR TITLE-ABS-KEY (brain AND lesion) OR TITLE-ABS-KEY (brain AND cancer) OR TITLE-ABS-KEY (brain AND neoplasm))) AND ((TITLE-ABS-KEY (white AND matter AND tract AND integrity) OR TITLE-ABS-KEY (white AND matter AND tract AND morphology) OR TITLE-ABS-KEY (white AND matter AND tract AND microstructure) OR TITLE-ABS-KEY (white AND matter AND tract AND topology) OR TITLE-ABS-KEY (white AND matter AND tract AND characterization) OR TITLE-ABS-KEY (white AND matter AND tract AND characterisation) OR TITLE-ABS-KEY (white AND matter AND criteria) OR TITLE-ABS-	973

	KEY (white AND matter AND tract AND alteration) OR TITLE-ABS-KEY (white AND matter AND tract AND changes))) View less	
WoS	((ALL=(diffusion tensor imaging) OR ALL=(diffusion tensor tractography) OR ALL=(diffusion tensor) OR ALL=(tractography)) OR ALL=(fiber tracking) OR ALL=(fibre tracking)) AND (ALL=(brain tumor (All Fields) or brain tumour (All Fields) or brain lesion (All Fields) or brain cancer (All Fields) or brain neoplasm (All Fields)) AND (ALL=(white matter tract integrity) OR ALL=(white matter tract morphology) OR ALL=(white matter tract microstructure) OR ALL=(white matter tract topology) OR ALL=(white matter tract characterization) AND ALL=(white matter tract characterisation) AND ALL=(white matter criteria) AND ALL=(white matter tract alteration) AND ALL=(white matter tract changes)))	881
	Total included duplicates	2775
	Total unique articles	1501
Phase 2		
Google scholar	Reference and citation from selected articles in Phase 1	2127
	Articles included	2
	Total selected articles for systematic review	25

Table S4: Quality Assessment of Selected Studies by NHLBI, NIH

S4.1.Quality assessment of case controlled study

	Criteria	Yu et al (2005)	Yen et al. (2009)	Schneider et al. (2021)	Zhang et al. (2016)	Itagiba et al (2010)	Deilami et al. (2015)
1	Was the research question or objective in this paper clearly stated and appropriate?	Y	Y	Y	Y	Y	Y
2	Was the study population clearly specified and defined?	Y	Y	Y	Y	Y	Y
3	Did the authors include a sample size justification?	N	Y	Y	Y	N	Y
4	Were controls selected or recruited from the same or similar population that gave rise to the cases (including the same timeframe)?	Y	Y	Y	Y	Y	Y
5	Were the definitions, inclusion and exclusion criteria, algorithms or processes used to identify or select cases and controls valid, reliable, and implemented consistently across all study participants?	Y	Y	Y	Y	Y	Y
6	Were the cases clearly defined and differentiated from controls?	Y	Y	Y	Y	CD	Y
7	If less than 100 percent of eligible cases and/or controls were selected for the study, were the cases and/or controls randomly selected from those eligible?	Y	N	N	Y	N	Y
8	Was there use of concurrent controls?	N	N	N	N	N	N
9	Were the investigators able to confirm that the exposure/risk occurred prior to the development of the condition or event that defined a participant as a case?	Y	Y	Y	Y	Y	Y
10	Were the measures of exposure/risk clearly defined, valid, reliable, and implemented consistently (including the same time period) across all study participants?	Y	Y	Y	Y	Y	Y
11	Were the assessors of exposure/risk blinded to the case or control status of participants?	Y	N	N	N	Y	N

12	Were key potential confounding variables measured and adjusted statistically in the analyses? If matching was used, did the investigators account for matching during study analysis?	Y	Y	N	Y	Y	Y

Yes, N; NO, Other (CD; cannot determine, NA; not applicable; NR; not reported

S4.2. Quality Assessment tool for Before – After (Per-Post) Studies With No Control Group

Criteria	Witwer et al (2002)	Field et al. (2004)	Lazar et al. (2006)	Chen et al. (2007)	Carvi y Nievas et al. (2010)	Kovanlikaya et al. (2009)	Bagadia et al. (2010)	Castellano et al. (2012)	Ibrahim et al. (2013)	Farshidfar et al. (2014)	Zhukov et al. (2016)	Dubey et al. (2018)	Yu et al. (2019)	Leroy et al. (2020)	Bakhshi et al. (2021)	Khan et al. (2019)	Shalan et al. (2021)	Wende et al. (2021)	Camins et al. 2022
Was the study question or objective clearly stated?	Y	Y	Y	Y	Y	Y	Y	Y	Y	Y	Y	Y	Y	Y	Y	Y	Y	Y	Y
Were eligibility/selection criteria for the study population prespecified and clearly described?	Y	Y	Y	Y	Y	Y	Y	Y	Y	Y	Y	Y	Y	Y	Y	Y	Y	Y	Y
Were the participants in the study representative of those who would be eligible for the test/service/intervention in the general or clinical population of interest?	Y	Y	Y	Y	Y	Y	Y	Y	Y	Y	Y	Y	Y	Y	Y	Y	Y	Y	Y
Were all eligible participants that met the prespecified entry criteria enrolled?	Y	Y	N	Y	Y	Y	Y	Y	Y	Y	Y	Y	Y	Y	Y	Y	Y	Y	Y
Was the sample size sufficiently large to provide confidence in the findings?	N	N	N	N	N	N	Y	Y	Y	N	N	N	N	N	Y	Y	N	N	N
Was the test/service/intervention clearly described and delivered consistently across the study population?	Y	Y	Y	Y	Y	Y	Y	Y	Y	Y	Y	Y	Y	Y	Y	Y	Y	Y	Y
Were the outcome measures prespecified, clearly defined, valid, reliable, and assessed consistently across all study participants?	Y	Y	N	Y	Y	Y	Y	Y	Y	Y	Y	Y	Y	Y	Y	Y	Y	Y	Y
Were the people assessing the outcomes blinded to the	N	N	N	N	N	N	Y	N	N	N	N	N	N	Y	Y	N	N	N	N

participants' exposures/interventions?																			
Was the loss to follow-up after baseline 20% or less? Were those lost to follow-up accounted for in the analysis?	Y	Y	Y	Y	Y	Y	Y	Y	Y	Y	Y	Y	Y	Y	Y	Y	Y	Y	Y
Did the statistical methods examine changes in outcome measures from before to after the intervention? Were statistical tests done that provided p values for the pre-to-post changes?	N	Y	Y	Y	Y	Y	Y	Y	Y	Y	Y	Y	Y	Y	Y	Y	Y	Y	Y
Were outcome measures of interest taken multiple times before the intervention and multiple times after the intervention (i.e., did they use an interrupted time-series design)?	N	N	N	N	Y	N	N	N	N	N	N	N	N	N	N	N	N	N	Y
If the intervention was conducted at a group level (e.g., a whole hospital, a community, etc.) did the statistical analysis take into account the use of individual-level data to determine effects at the group level?	Y	Y	Y	Y	Y	Y	Y	N	N	Y	Y	Y	Y	Y	Y	N	Y	N	Y

Yes, N; NO, Other (CD; cannot determine, NA; not applicable; NR; not reported
